# Schizophrenia Detection and Classification by Advanced Analysis of EEG Recordings Using a Single Electrode Approach

**DOI:** 10.1371/journal.pone.0123033

**Published:** 2015-04-02

**Authors:** Zack Dvey-Aharon, Noa Fogelson, Avi Peled, Nathan Intrator

**Affiliations:** 1 Blavatnik School of Computer Science, Tel-Aviv University, Tel-Aviv, Israel; 2 The Joseph Sagol Neuroscience Center, Sheba Medical Center, Tel Hashomer, Israel; 3 Department of Psychology, University of A Coruña, La Coruña, Spain; 4 Ruth and Bruce Rappaport Faculty of Medicine, Technion, Israel; 5 Institute for Psychiatric Studies, Sha’ar Menashe Mental Health Center, Hadera, Israel; Center for BrainHealth, University of Texas at Dallas, UNITED STATES

## Abstract

Electroencephalographic (EEG) analysis has emerged as a powerful tool for brain state interpretation and diagnosis, but not for the diagnosis of mental disorders; this may be explained by its low spatial resolution or depth sensitivity. This paper concerns the diagnosis of schizophrenia using EEG, which currently suffers from several cardinal problems: it heavily depends on assumptions, conditions and prior knowledge regarding the patient. Additionally, the diagnostic experiments take hours, and the accuracy of the analysis is low or unreliable. This article presents the “TFFO” (Time-Frequency transformation followed by Feature-Optimization), a novel approach for schizophrenia detection showing great success in classification accuracy with no false positives. The methodology is designed for single electrode recording, and it attempts to make the data acquisition process feasible and quick for most patients.

## Introduction

Electroencephalography (EEG) is the measurement of electrical activity along the scalp, mostly via manually placed electrodes at different locations or a headset device designed for that purpose.

EEG is increasingly used as a low-resolution diagnosis tool for general cognitive activity and as an indicator of the current brain state at a given time due to its effectiveness as a mobile quick-setup recordable tool. Past studies using EEG have included emotion detection [1;2], cognitive state determination [3;4], evaluation of activity area control based devices [5], analysis of epileptic seizures [6] and many other related methods. Due to a significant lack of signal sensitivity compared to other tools such as fMRI, EEG has not emerged as an efficient or accurate input method for the detection and analysis of mental illnesses. State of the art computational studies have suggested that mostly changes in functional connectivity are seen in schizophrenia patients [7], and significant differences in theta-frequency activity are evident as well [8]. Past studies to diagnose schizophrenia using EEG have focused on classic ERP (Event Related Potential) analysis, in particular examining properties around N100 and P300 [9;10] and a recent study has also suggested to add a machine learning mechanism to analyze a few P300 attributes [11] showing significant accuracy in classification.

In addition, conducting a full-scale EEG-based analysis requires, in most cases, recording from patients for hours. Recording from subjects potentially suspected of mental illness might not be feasible in many cases, or may be an arduous effort—especially when using a complex multi-electrode setup.

The challenge is to find a reliable discriminating technology to discern the responses of the healthy subjects and schizophrenia patients using limited data. In the scope of this study, the chosen approach is to propose such a method based on the input of a single electrode and using less than one minute of recording data from a subject to achieve an accurate classification result.

In this paper, we describe a novel methodology and illustrate the clinical experiment performed to detect the presence of schizophrenia. The results indicate an accurate classification of healthy individuals and schizophrenia patients. The results also indicate that robust discrimination can be achieved by the analysis of approximately one minute of data with only one to three electrodes.

## Methodology

### Problem Description

The current paper examines the question of whether there is sufficient information in EEG data, which are collected over a short time—less than a minute—to differentiate between healthy subjects and subjects who have been diagnosed with schizophrenia and are receiving relevant prescribed medication. In particular, we are concerned with the question of whether data from a single electrode can produce such a distinction. A second research question is whether the severity of the disease, as measured by the number of hospitalizations or other known markers, is correlated with the results of the model (although these data were not used to form the model).

### Methods

The general theme of the proposed method relies on the assumption that a given dataset of two populations holds discriminating information that can be easily acquired and measured using the EEG tool, and the challenge is finding the significant components of the data that can contribute the most to the desired discrimination.

Given such data, an optimization analysis was used to emphasize the statistically significant difference in the responses between the two populations. The optimization was performed using several parameters:

1. Response time: finding the most discriminating interval following a stimulus trigger.
2. Acquisition time: given a collection of stimuli, the analysis examining how the discrimination power is spread over the course of stimuli.
3. Frequency bands: identifying which frequency bands are most relevant for the process of discriminating the data.
4. Electrode: the electrode(s) from which the data are taken.
5. The EEG signal is acquired as a time series and is also converted into a time/frequency representation using the Stockwell transform. Another layer of optimization is to extract the most significant components from the Stockwell transform representation.

In the specific case of schizophrenia, the assumption is that the expectation for an object (in a sequence of objects) may be different between healthy subjects and schizophrenia patients. Therefore, specific experiments exploit this assumption by creating such an expectation and measuring the response of both populations to this stimulus type.

**Fig 1:**
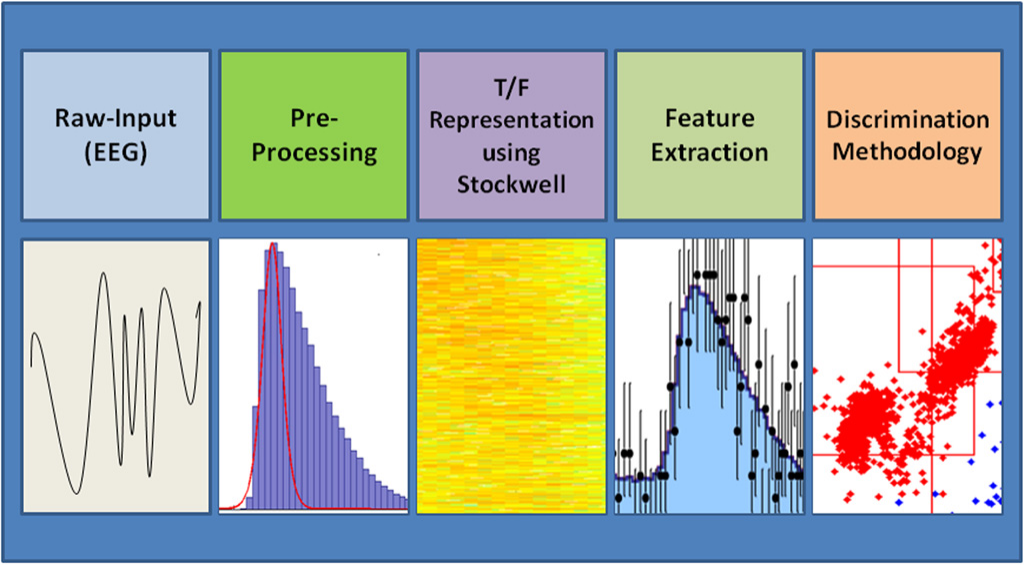
Phases of the TFFO methodology.

The proposed discrimination system is based on a Time-Frequency transformation followed by Feature-Optimization (“TFFO”). It is composed of four stages: (a) Raw-Data Preprocessing, performing several preprocessing tasks and breaking the raw signals into relevant intervals, (b) Transformation of the EEG signal into a time/frequency representation via the Stockwell transform [12], (c) Feature extraction from the time/frequency representation, and (d) Discrimination of specific time frames following a given set of stimuli between the time/frequency matrix representations of the healthy subjects and the schizophrenia patients. Fig 1 below presents the four stages of the methodology.

EEG data from a previously reported study [13] were used. This study provided evidence for contextual processing deficits in patients with schizophrenia by demonstrating alterations in the neural correlates of local contextual processing. As will be described further below, the acquired data are a recorded set of signals triggered by the stimuli and followed by mouse clicks performed by the subjects during a computer simulation (game) in which graphic triangles appear on the screen and the subjects (players) are asked to press the button in certain cases according to the orientation of the triangles.

EEG recording was performed with a 64 electrode array using the ActiveTwo system (Biosemi, The Netherlands). External electrodes above and below the right eye monitored vertical eye movements, and electrodes placed laterally to the left and right eyes monitored horizontal eye movements. Signals were amplified and digitized at 512 Hz.

The four stages were applied to data from each electrode separately to enable comparison between single-electrode discrimination power.

In the preprocessing phase, a band-pass filter of 0.1–30 Hz was applied, and the total energy was normalized. The data were separated into frames of 1.2 seconds, with each interval starting 200 ms prior to the appearance of the ‘P’ shaped stimuli. The ‘P’ stimulus produced the best discrimination between healthy subjects and patients diagnosed with schizophrenia, similar to a previous study [13].

Eye blinks and other artifacts were removed using ICA [14]. Epochs containing misses (no button press 150–1150 ms post-stimulus onset) and eye saccades were excluded from further analysis. EEG epochs with amplitudes of more than 75 μV at any electrode were also excluded.

After the pre-processing stage, data were separated into two different datasets, train and test, as specified below. The process described below was performed on the train dataset, and the achieved parameters were tested using the test dataset. Time windows of 100/500 ms (transitioned by 25 ms/50 ms; 50% overlap) were used to search for the most significant sub-interval for analysis. For each window, additional stages were performed using only a given sub-interval of the original full 1.2 second window. The time windows were used to obtain further validation of the discrimination power of windows that contain P300-like features. Examining the 1.2 second interval before and after each stimulus is similar to the general concept of ERP. The main difference between standard ERP and the methodology presented here is the use of Stockwell features instead of the raw-signal representations and the addition of a learning mechanism on top of the features to obtain optimal discrimination. A basic time-frequency transformation without an additional learning mechanism was suggested in a previous study [15].

### T/F representation

From the Stockwell representation of the data, frequency-band windows of 5 Hz/10 Hz (transitioned by 1 Hz/2 Hz; 80% overlap) were used to search for a maximum variance band. The redundancy in this frequency representation produced better discriminative features than less redundant frequency representations as well as standard EEG frequency bands (Berger [16]). The following steps were performed using only the sub-interval window in the chosen frequency band. The discrimination was calculated on a varying sequential number of stimuli chosen from the full set of stimuli. The sequence of stimuli was chosen from the first and last stimulus.

The following process was performed in each iteration of the parameter enumeration described above. Given a subset of events for each subject, a window [*a*, *b*] was filtered for a set of frequencies {f} composed of the average of the subjects’ signals according to parameter K. The procedure was followed by performing an implementation of the Stockwell transform [12], known as a generalization of the short-time Fourier Transform and denoted by

$$TF\left(x\right)\;=\;S_{x}\left(t,\;f\right)\;=\;\int_{a}^{b}x\left(\tau \right)\left|f\right|e^{-\pi {\left(t-\tau \right)}^{2}f^{2}}e^{-j2\pi f\tau }d\tau$$,

derived as phase correction of the continuous wavelet transform.

The Stockwell transform produces a vector *x*_*i*_ *i = 1…*, *n* of features for each frame. The Euclidian distance between such vectors was used for the creation of a K-nearest neighbor classifier, and score denoted by

$$distance_{i}\;=\;\sum \underset{k}{\min}||x_{i},x_{j}||\;=\;\sum \underset{k}{min}||S_{i}\left(t,f\right)-S_{j}(t,f)||,i\neq j$$

Cross-validation was performed using ‘leave one out’ [17]; prediction of the class of each tested subject was achieved by training with the full remaining dataset, excluding one patient’s data each time, and using this ‘left out’ data as a test set. Test result is therefore given as average on all subjects, where each subject was tested once on a model trained without that subject. [18] Mathematically, matrix F presents the Stockwell features per class (healthy and schizophrenia diagnosed patients). The regressor for testing a subject’s data was built after temporarily excluding all vectors *x*_*i*_ *i = 1…*, *n* that were recorded from the subject.

Given the limited number of subjects in the dataset, this technique allowed effective validation and guarded against errors in the data. For optimization purposes, a parameter K for the number of voting neighbors to be checked was enumerated in the range: $1\leq K\leq \frac{\left|events\right|}{2}$ for best classification. In the case that K is even, the closest neighbor has a casting vote. Lastly, regression for patient severity prediction was performed from the acquired time-frequency representation distance measurement. Ridge linear regression including a penalty to reduce overfitting was used.

Accuracy for each classification was computed by dividing correct classifications by the total number of analyzed subsets of patient data.

The execution of the proposed methodology described above on the EEG data provided us with a high-dimensional matrix; each dimension of the matrix is a parameter of the learning process, such as the electrode used, location of the window used from each recording, or the frequency band used as a filter. Each entry in the matrix is a set of cross-validated prediction results referring to the success in predicting the different test datasets given the parameters that are represented by the coordinates of the entry.

Examination of this matrix can not only reveal what combinations of the possible configurations featured the best prediction results but also show for each dimension what values achieved the highest scores on average (figuring other configurations into the calculation as well), so that more specific conclusions regarding the parameters and their connection to the physical attributes can be made.

In addition to the binary classification task, a ridge regression was performed to search for a correlation between the acquired distance feature and general medical properties (such as amount of medicine taken by the patient, number of recorded hospitalizations, and number of positive/negative symptoms) of the diagnosed subjects because it has been proven to be a powerful tool for time-frequency EEG-based features [19;20].

### Data Description

The complete data collection process and experimental description are as described in previous studies [13;21;22] and are further specified below.

The experiment is based on objects that appear on a computer screen. It includes the creation of anticipation for a specific pattern at a specific angle and then measures the responses of the subjects.

A full data description appears in a previous study [13]. For completeness, we describe here the main features of the data.

The experiment data recording set was obtained from a group of 50 patients, 25 healthy subjects with no prior indication of mental illness or abnormal behavior and 25 subjects who had been diagnosed with schizophrenia, hospitalized and treated with medication. The study was approved by the ethics committees of the University of A Coruna, Spain, and Sha’ar Menashe Mental Health Center in Hadera, Israel. All subjects signed a written consent form. Patients were diagnosed with schizophrenia according to the Structured Clinical Interview for DMS-IV-Tr and were rated for symptom severity using the Positive (SAPS) and Negative (SANS) Syndrome Scale [23]. We note that during the recording phase, the schizophrenia patients were on their regular medication. All recordings took place in the morning (10:00–12:00).

The stimuli consisted of black triangles on a gray background (triangles facing left, upwards and right, at 90 degree increments). In each block, a total of 78 stimuli were presented for 150 ms each with an inter-stimulus interval (ISI) of 1 second. Stimuli blocks were a mixture of either randomized sequences of standards or sequences including a three-standard predictive sequence. The predictive sequence always consisted of the three types of triangles facing left, up and right, always in that order. We focused our analysis on data obtained from the predictive sequences (“P” stimuli) because they are considered to be a significant indication of schizophrenia [13;21;22].

## Results and Discussion

### I. General methodology accuracy as a function of electrode positioning

**Fig 2:**
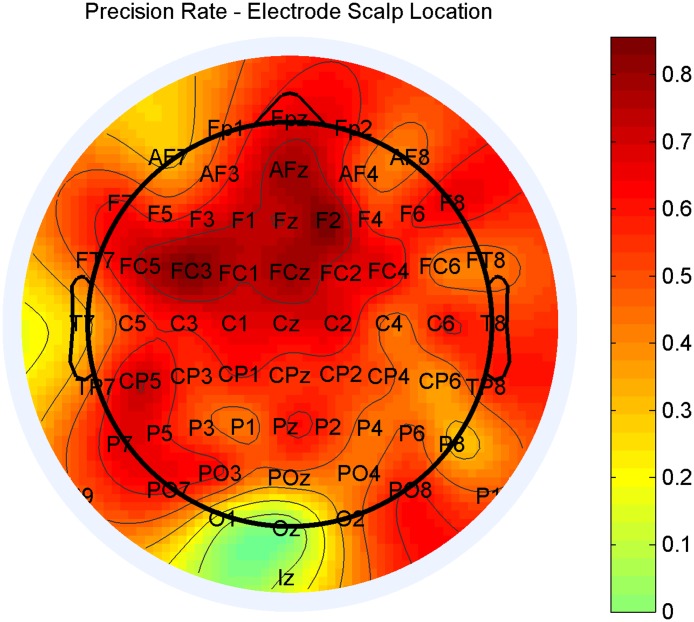
Accuracy of prediction for each electrode. The frontal-central scalp area showed the most significant variance between healthy subjects and patients diagnosed with schizophrenia.

The results indicated a high accuracy for classification of the data, with a prediction accuracy of the top 5 distinctive electrodes between 91.5% and 93.9% with the best electrode—F2. The most accurate electrodes with the best distinction between the two classes were F2 and FC3, followed by AFz, FCz and FC5, as seen in Fig 2 below. One can see the most distinctive electrodes geographically close to one another and are located in a region previously found to be relevant to schizophrenia [22]. while F2 showed strong results, similar statistical discrimination results were obtained with other electrodes such as FC3, with which there is no correlation to visual input. Additionally, ICA should have completely removed the blink effect from the signal, or at least prevented it from being a significant factor even in the visual-area-based electrodes.

The discrimination accuracy of the presented methodology was compared with both latency-based approaches and subject reaction time [23]. Basic comparison to subject latency analysis was performed by (a) calculating the distance to peak energy amplitude and clustering using the K-Means [“PE-Latency”] for each stimulus and (b) repeating the post-feature extraction methodology phases while replacing the time-frequency features with the distribution of the energy peak location in the P300 area [“P300-RF-Latency”] and performing “RT ANOVA”—a reaction time-based approach for the discrimination of healthy subjects and schizophrenia patients [22]. The discrimination results are presented in Table 1 below.

**Table 1:** Discrimination accuracy per tested methodology.

Methodology	Discrimination Accuracy Rate	Specificity Rate	Sensitivity Rate	Significance P-Value
TFFO (proposed here)	88.7%±4%	100%	77.4%±7.1%	0.0078
P300 Derived Features—Reaction Time [22]	73.9%±4.3%	87.3%±3.1%	60.5±5.4%	0.043
P300-RF-Latency	68.1%±4.8%	82%±3.9%	54.2±5.5%	0.0919
PE-Latency [23]	64.5%±5.5%	74.2%±4.2%	58.7±7.2%	0.1746

The accuracy achieved by the TFFO methodology is significantly better than other reaction latency related implementations, proving the added value of the analysis beyond latency differences between healthy subjects and schizophrenia patients (P-Value<1%). Moreover, the results obtained did not include any false positives, i.e., no healthy subject was classified as a schizophrenia patient. Therefore, it is desirable in such problems to have high specificity rather than high sensitivity. Generally, frontal area EEG input correlation to cognitive-related features and mental disorders is evident. Recent work in patients with schizophrenia [13;22;24] and Parkinson’s disease [25] has shown high input variance in that segment. Such evident correlations can explain the obtained results.

### II. Representation of signal variance over time

**Fig 3:**
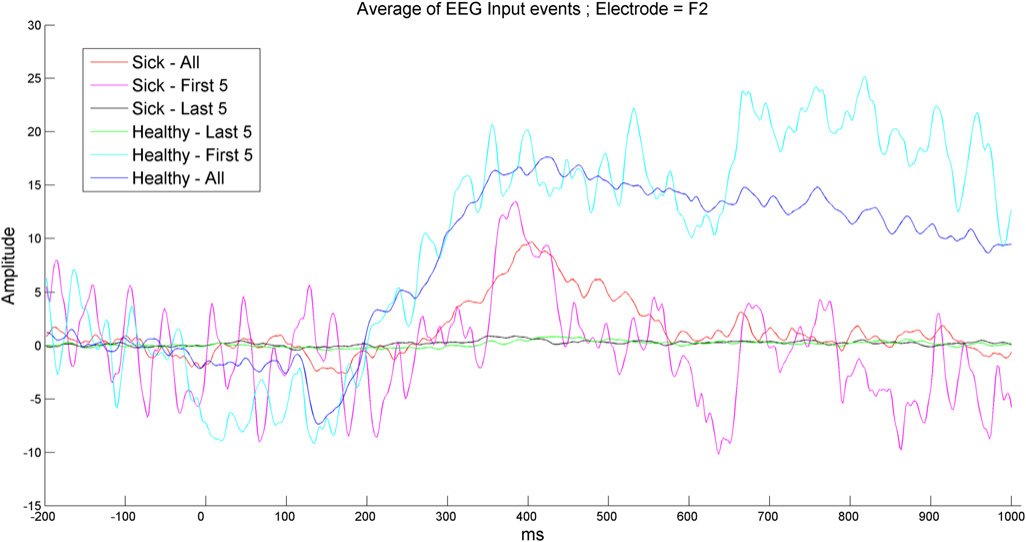
Average signal amplitude for healthy subjects and patients diagnosed with schizophrenia within total, early or late sets of events. The first 5 reactions to the stimulations result in peak responses; the variance between healthy subjects and patients diagnosed with schizophrenia is larger than the variance obtained by the average amplitude of 8 entire event sets. Additionally, the last 5 events of both healthy subjects and patients diagnosed with schizophrenia resulted in almost no reaction and almost zero variance between the two classes.

The results showed that the reaction of both diagnosed patients and healthy subjects to non-early stimulations is between weak and non-existent. As seen in Fig 3 below, which is taken from a significantly discriminating electrode obtained from part I, the first several recordings possessed the most variance, which was significantly higher than the variance obtained in the general average. Therefore, better discrimination can be achieved with less data by having a significantly quicker acquisition process.

### III. Methodology accuracy as a function of events obtained from patients

**Fig 4:**
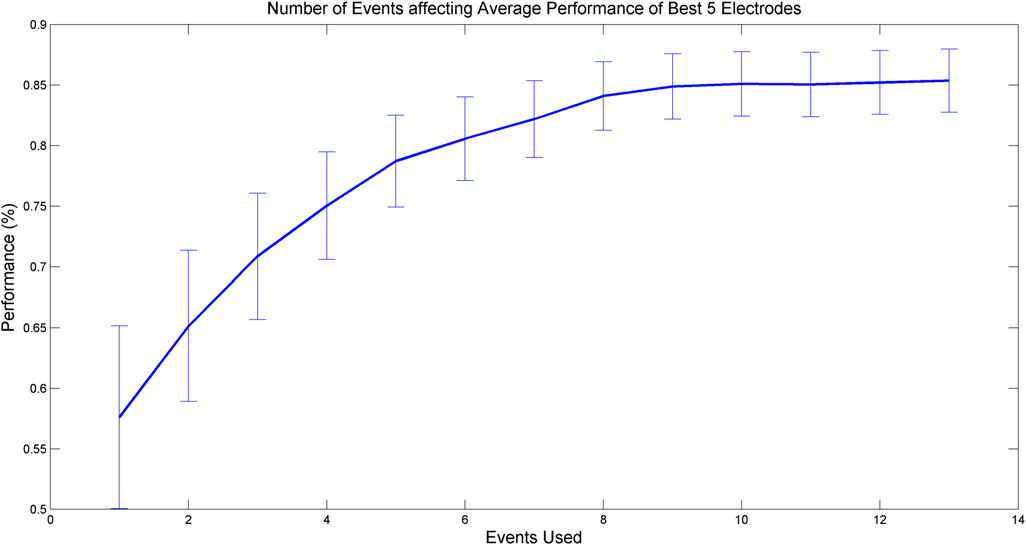
Accuracy of prediction as a function of events obtained from each subject’s initial recording of stimulation events. By using only the first 7 or 8 events from each subject, the prediction accuracy of the methodology is close to optimum.

As indicated in the above results, several initial stimuli possessed the most discriminating variance between the two classes of subjects. The resulting optimal learning parameters show that achieving the best separation with the first 7 to 8 events in each subject results in optimal accuracy. This can be clearly seen in Fig 4 below, which plots the percentage of prediction accuracy as a function of events taken from the beginning of the recordings for each subject.

### IV. Methodology accuracy as a function of the internal time window of recorded stimulations

**Fig 5:**
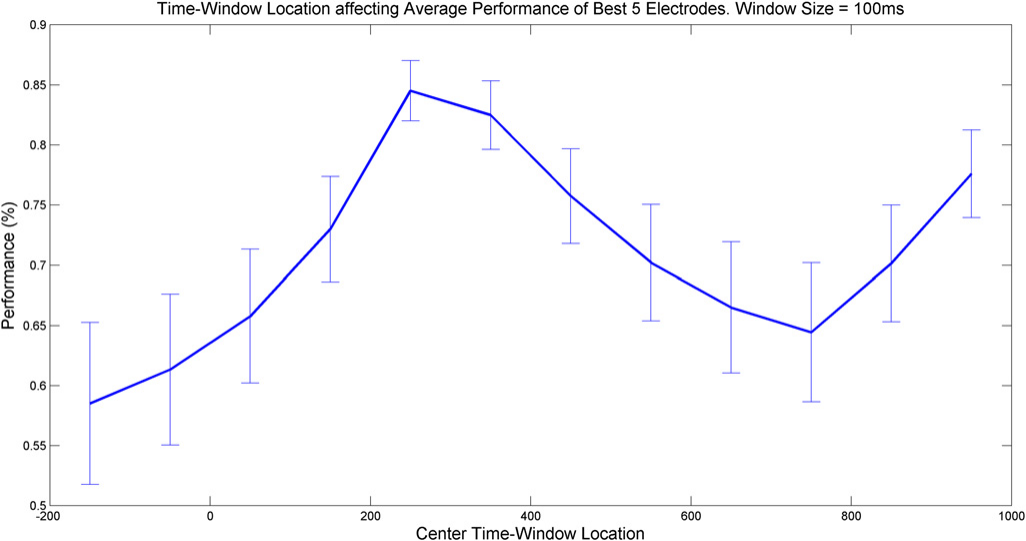
Accuracy of methodology prediction as a function of time window within each stimulus recording. The most variance (or local peaks of methodology accuracy) is best achieved with a ~300 ms window placed in the recording window post-stimulus.

The results for the optimal [a, b] window within the stimulus recording interval (of each appearance of the ‘P’ stimulus) showed that the most accurate predictions can be achieved at intervals of (a) 200–300 ms post-stimulation and (b) 900–950 ms post-stimulation. These results are presented in Fig 5 below. The interval in (a) can be explained by latency in the analysis process in the frontal area located near the P300 recording area, which is evident from significant visual-related stimuli [26]. The interval in (b) can be explained by the latency of recovery from visual analysis and prediction of the brain activity, which is evident from differences in subjects with mental disorders [26;27;28].

### V. Methodology accuracy as a function of the selected frequency band

**Fig 6:**
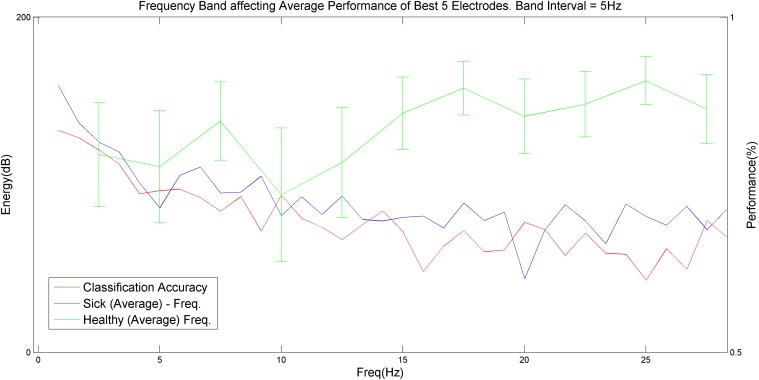
Accuracy of methodology prediction as a function of the selected frequency band. The most variance (or local peaks of methodology accuracy) is best achieved using a frequency interval of 15–20 Hz or 22.5–27.5 Hz (mostly Beta2 frequencies). It is clear that the Delta, Theta and Alpha frequency bands possess less discrimination power.

The results for the optimal frequency band showed that the most accurate prediction can be achieved using Beta2 band frequencies, specifically with 15–20 Hz and 22.5–27.5 Hz bands, which achieved maximal discrimination between healthy subjects and schizophrenia patients. This finding is clearly demonstrated in Fig 6 below.

### VI. Regression-based correlation between disorder severity and methodology feature extraction

By performing a regression-based correlation between the schizophrenia patients’ extracted features and predicting their documented severity, a strong physiological justification for the validity of the features was acquired. We transformed the number of documented hospitalizations of each patient into an exponential scale (1, 2, 3–4, 5–7, 8–15, 16+) and attempted to predict the transformed hospitalization measure using the transformed Euclidian distance from each patient to the average features of the healthy group. We found that 71.7% (with the best classifying electrode, F2) of the variance of the transformed measurement is accounted for. These findings support the claim that the TFFO methodology predicts the severity of the disease rather than the medication taken for its treatment because the medication dosages were not correlated with the number of hospitalizations, and a similar attempt to correlate diagnosed patients’ dosages to the disorder severity showed only minor correlation.

By sorting the patients according to the Euclidian distance from the averaged features of the healthy subjects group, we found that (a) 4 out of the 5 patients with the most negative symptoms were in the ‘severe half’ of the sorted feature; (b) 4 out of 5 of the patients with the most hospitalizations were in the ‘severe half’ of the sorted feature; and (c) 4 out of 5 of the patients taking the highest dose-wise medication load were in the ‘severe half’ of the sorted feature. Therefore, our results indicate the strong and direct connection between our discriminating features and the disorder parameters and suggest their ability to act as discriminators of the disease and its severity.

## Conclusions and Further Work

We presented a novel methodology for correlating EEG features and the schizophrenia condition. We demonstrated that these features can be detected using a small number (up to three) of well-located electrodes and acquired in less than a minute of recording time for most patients. This holds great potential for future medical diagnosis, monitoring and neurofeedback, enabling patients to be released from the hospital earlier and monitored for the use and effectiveness of their medications, and it may be utilized to reduce the severity of their symptoms using neurofeedback.

Future work can also focus on expanding the methodology for detecting additional types of mental disorders or illnesses or for performing a general diagnosis by acquiring any abnormal mental indications, which can transform detection methods into a reliable set of processes.

Additionally, the methodology can be further improved to achieve a higher accuracy and to diagnose illness severity in a precise manner. It is possible that a machine learning model will assist in this process and replace traditional diagnosis procedures in the future.

## References

[1] Petrantonakis PC, Hadjileontiadis LJ. Emotion Recognition From EEG Using Higher Order Crossings, Information Technology in Bio, 2010;14: 186–197. 10.1109/TITB.2009.2034649 19858033

[2] Oude DB. EEG-based Emotion Recognition—The Influence of Visual and Auditory Stimuli, 2007, Available: http://hmi.ewi.utwente.nl/verslagen/capita-selecta/CS-Oude_Bos-Danny.pdf

[3] Zhdanov A, Hendler T, Ungerleider L, Intrator N. Machine Learning Framework for Inferring Cognitive State from Magnetoencephalographic (MEG) Signals, Advances in cognitive neurodynamics, ICCN, 2007;3: 393–397.

[4] Zhdanov A, Hendler T, Ungerleider L, Intrator N. Inferring Functional Brain States using Temporal Evolution of Regularized Classifiers, Intell Neuroscience, 2007. 10.1155/2007/52609 PMC2266829 18350130

[5] Lotte F, Congedo M, Lécuyer A, Lamarche F, Arnaldi B. A review of classifcation algorithms for eeg-based brain-computer interfaces—Journal of Neural Engineering, 2007: 1–13. 17409472 10.1088/1741-2560/4/2/R01

[6] Peled A, Geva A, Kermen W, Blankfeld H, Estfandiarfard R, Nordahl T. Functional Connectivity and Working Memory in Schizophrenia: An EEG Study, International Journal of Neuroscience, 2001;106: 47–61. 11264908 10.3109/00207450109149737

[7] Koenig T, Lehmann D, Saito N, Kuginuki T, Kinoshita T, Koukkou M. Decreased functional connectivity of EEG theta-frequency activity in first-episode, neuroleptic-naïve patients with schizophrenia: preliminary results, Schizophrenia Research, 2001;50: 55–60. 11378314 10.1016/s0920-9964(00)00154-7

[8] Geva A, Kerem D. Forecasting Generalized Epileptic Seizures from the EEG Signal by Wavelet Analysis and Dynamic Unsupervised Fuzzy Clustering, IEEE Transactions of Medical Engineering, 1998;45(10): 1205–1216. 9775534 10.1109/10.720198

[9] Neuhaus A, Popescu FC, Grozea C, Hahn E, Hahn C, Opgen-Rhein C, et al, Single-Subject Classification of Schizophrenia by Event-Related Potentials during Selective Attention, NeuroImage, 2011;55(2): 514–521. 10.1016/j.neuroimage.2010.12.038 21182969

[10] Neuhaus A, Popescu FC, Rentzsch J, Galliant J. Critical Evaluation of Auditory Event-Related Potential Deficits in Schizophrenia: Evidence from Large-Scale Single-Subject Pattern Classification, Schizophrenia Bulletin, 2013, 10.1093/schbul/sbt151 PMC4133667 24150041

[11] Laton J, Schependom JV, Gielen J, Decoster J, Moons T, Keyser JD, et al, Single Subject Classification of Schizophrenia Patients based on a Combination of Oddball and Mismatch Evoked Potential Paradigms, Journal of the Neurological Sciences, 2014;347(1): 262–267. 25454645 10.1016/j.jns.2014.10.015

[12] Stockwell RG, Mansinha L, Lowe R. Localization of the Complex Spectrum: the S Transform, IEEE Trans. Signal Processing, 1996; 44(4): 998–1001.

[13] Fogelson N, Shah M, Scabini D, Knight RT. Prefrontal cortex is critical for contextual processing: Evidence from brain lesions. Brain, 2009;132: 3002–3010. 10.1093/brain/awp230 19713281 PMC2768662

[14] Yandong L, Zhongwei M, Lu W, Li Y. Automatic Removal of the Eye Blink Artifact from EEG using an ICA-based Template Matching Approach, Physiological Measurement, 2006;27: 425–436. 16537983 10.1088/0967-3334/27/4/008

[15] Roach BJ, Mathalon DH. Event-Related EEG Time-Frequency Analysis: An Overview of Measures and an Analysis of Early Gamma Band Phase Locking in Schizophrenia, Schizophrenia Bulletin, 2008;34(5): 907–926. 10.1093/schbul/sbn093 18684772 PMC2632478

[16] Berger H. Psyche 6, 1940

[17] Halkidi M, Vazirgiannis M. Clustering Validity Assessment Using Multi Representatives, SETN, 2002: 237–248.

[18] Beer D, Kardia S, Haung C, Giordano T, Levin A, Misek D, et al, Gene-Expression Profiles Predict Survival of Patients with Lung Adenocarcinoma, Nature Madicine, 2002;8: 816–824. 12118244 10.1038/nm733

[19] Podlipski I, Ben Simon E, Hendler T, Intrator N. Robust Modeling on Optimized EEG Bands for Functional Brain State Inference, Journal Of NeuroScience Methods, 2011;203: 377–385. 10.1016/j.jneumeth.2011.10.015 22044846

[20] Tomioka R, Aihara K, Müller R. Logistic Regression for Single Trial EEG Classification, Advances in neural information processing systems, 2007;19: 1377–84.

[21] Fogelson N, Shah M, Bonnet-Brihault F, Knight RT. Electrophysiological evidence for aging effects on local contextual processing. Cortex, 2010;46: 498–506. 10.1016/j.cortex.2009.05.007 19559410 PMC2826523

[22] Fogelson N, Ribolsi M, Fernandez-del-Olmo M, Rubino IA, Romeo D, Koch G, et al, Neural correlates of local contextual processing deficits in schizophrenic patients. Psychophysiology, 2011;48: 1217–1226. 10.1111/j.1469-8986.2011.01195.x 21446992

[23] Andreasen NC, Olsen S. Negative and positive Schizophrenia: Definition and validation. Archives of General Psychiatry, 1982;39: 789–794. 7165478 10.1001/archpsyc.1982.04290070025006

[24] Fenton G, Fenwick P, Dollimore J, Dunn T, Hirsch S. EEG Spectral Analysis in Schizophrenia, The British Journal of Psychiatry, 1980, 10.1192/bjp.136.5.445 7388249

[25] Fogelson N, Fernandez-del-Olmo D, Santos-Garcia D. Contextual processing deficits in Parkinson´s disease: Role of the frontostriatal system, Clinical Neurophysiology, 2011;122: 539–545. 10.1016/j.clinph.2010.07.017 20709594

[26] Carretié L, Iglesias J, García T, Ballesteros M. N300, P300 and the emotional processing of visual stimuli, Electroencephalography and Clinical Neurophysiology, 1997;103: 298–303. 9277632 10.1016/s0013-4694(96)96565-7

[27] Andreasen NC, Olsen S. Meta Analysis of P300 and Schizophrenia: Patients, Paradigms and Practical Implications, Psychophysiology, 2003;40: 684–701. 14696723 10.1111/1469-8986.00070

[28] Bestelmeyer P, Philips L, Crombie C, Benson P, St. Clair D. The P300 as a Possible Endophenotype for Schizophrenia and Bipolar Disorder: Evidence from twin and patient studies, Psychiatry Research, 2009;169: 212–219. 10.1016/j.psychres.2008.06.035 19748132

